# Genome-Wide Identification and Characterization of JAZ Protein Family in Two *Petunia* Progenitors

**DOI:** 10.3390/plants8070203

**Published:** 2019-07-03

**Authors:** Shaoze Tian, Siyu Liu, Yu Wang, Kun Wang, Chaoqun Yin, Yuanzheng Yue, Huirong Hu

**Affiliations:** 1 Key Laboratory of Urban Agriculture in Central China, Ministry of Agriculture and Rural Affairs, Key Laboratory of Horticultural Plant Biology, Ministry of Education, College of Horticulture and Forestry Sciences, Huazhong Agricultural University, Wuhan 430070, China; 2 Key Laboratory of Landscape Architecture, Jiangsu Province, College of Landscape Architecture, Nanjing Forestry University, Nanjing 210037, China

**Keywords:** JAZ, jasmonate, petunia, phylogenetic analysis, qRT-PCR, anther, MYC2

## Abstract

Jasmonate ZIM-domain (JAZ) family proteins are the key repressors in the jasmonate signaling pathway and play crucial roles in plant development, defenses, and responses to stresses. However, our knowledge about the JAZ protein family in petunia is limited. This research respectively identified 12 and 16 JAZ proteins in two *Petunia* progenitors, *Petunia axillaris* and *Petunia inflata*. Phylogenetic analysis showed that the 28 proteins could be divided into four groups (Groups A–D) and further classified into six subgroups (A1, A2, B1, B3, C, and D1); members in the same subgroup shared some similarities in motif composition and sequence structure. The Ka/Ks ratios of seven paralogous pairs were less than one, suggesting the petunia JAZ family might have principally undergone purifying selection. Quantitative real-time PCR (qRT-PCR) analysis revealed that *PaJAZ* genes presented differential expression patterns during the development of flower bud and anther in petunia, and the expression of *PaJAZ5*, *9*, *12* genes was generally up-regulated after MeJA treatment. Subcellular localization assays demonstrated that proteins PaJAZ5, 9, 12 were localized in nucleus. Yeast two hybrid (Y2H) elucidated most PaJAZ proteins (PaJAZ1-7, 9, 12) might interact with transcription factor MYC2. This study provides insights for further investigation of functional analysis in petunia JAZ family proteins.

## 1. Introduction

The oxylipin-derived phytohormone jasmonate (JA) regulates growth, development, secondary metabolism, and stress responses during the plant life cycle [1,2,3,4,5,6]. The (+)-7-iso-Jasmonoyl-L-isoleucine (JA-Ile), which has a structure greatly similar to coronatine, is the endogenous bioactive form of the hormone [7], and the biosynthesis of JA-Ile relies on the reduction of 12-oxo-phytodienoic acid (OPDA), the JA precursor, which can be reduced by OPDA reductase 3 (OPR3) [2]. Recently, an OPR3-independent pathway to synthesize bioactive JA was discovered. OPDA could enter into another pathway to produce a direct precursor 4,5-didehydro-JA for JA-Ile synthesis in the absence of OPR3 [8]. Previous research elucidated that JAZ proteins, as the repressors, could play crucial roles in JA signaling pathways [9,10,11]. Without the JA stimulation, TOPLESS (TPL) or TPL-related proteins (TRPs) could be recruited by JAZ proteins via the adapter protein NINJA to form the co-repressors complex [12] and further interact with a variety of downstream transcription factors to suppress diverse JA responses [3,6]. For example, the transcription factor MYC2 is a key regulator of the JA signaling pathway and has been verified to be inhibited by a series of JAZ proteins [13,14]. It has been reported that the JA receptor is a co-receptor complex composed of the F-box protein COI1, the JAZ repressor, and inositol pentakisphosphate [15]. In the presence of bioactive JA signal, the co-receptor COI1-JAZ facilitates the ubiquitination and the degradation of JAZ repressors by forming the SCF^COI1^ E3 ubiquitin ligase complexes in the 26S proteasome pathway, thereby leading to the release of several transcription factors and the activation of various JA responses [9,10,15,16]. The JAZ proteins act as two mutually constrained and highly conserved roles at the mechanism level; one is the transcriptional suppression and another is the signal perception, thereby coordinating the balance between development and defense [6,17,18]. In addition, JA and other plant phytohormones (gibberellic acid, ethylene and salicylic acid, etc.) display synergistic or antagonistic activities in regulating many aspects of plant growth and stress responses [19,20,21], and JAZ proteins play central roles in such activities to constitute the key node for crosstalk with different developmental and stress-response signaling pathways [6]. The complex crosstalk network between JA and other phytohormones may conceivably provide a considerate system that is essential for plants to adjust themselves to the continuously changed environmental conditions [22].

The JAZ protein family belongs to the plant-specific TIFY superfamily, which includes the other three members, namely, ZIM-like (ZML), TIFY, and PEAPOD (PPD) families [23,24,25]. There are two highly conserved functional domains, TIFY (also called ZIM) and Jas (also called CCT_2), characterized in JAZ family proteins, which is distinguished from the others [9]. The TIFY domain usually consists of 28 amino acids containing a core “TIF[F/Y]XG” motif [23], which is near the N-terminus of the JAZ protein sequences, and the structure prediction analysis shows the TIFY domain may form a secondary α-α-β fold [24]. The Jas domain, possessing the consensus sequence “SLX_2_FX_2_KRX_2_RX_5_PY”, is another defining trait of JAZ proteins closed to the C-terminus [9,10,15]. Both the TIFY and the Jas domains are reported to be necessary for JAZ proteins to function in the JA signaling pathway. The TIFY domain mediates hetero- and homomeric interactions between JAZ proteins regardless of the presence of hormones [26,27]. In addition, the interaction between JAZ proteins and the NINJA-TPL complex is also dependent on the TIFY domain [12]. The Jas domain, as the connecter, mediates the direct interaction with both COI1 and bHLH transcription factors, resulting in the inhibition of the transcriptional activities. Lacking the Jas domain is found to cause insensitivity to bioactive JA and exhibit constitutive repression of JA signaling [28,29,30]. Furthermore, it has been reported that the Jas domain can form an α-helix, which replaces the helix structure of the MYC3 N-terminus and becomes a part of the MYC N-terminal fold, which also leads to the inhibition of transcriptional activity [31].

JAZ family proteins have been reported in many plants, for instance, 13, 15, 14, 13, and 11 JAZ proteins have been identified in *Arabidopsis thaliana* [9,10,32], *Oryza sativa* [33], *Triticum aestivum* [34,35], *Solanum lycopersicum* [36], and *Vitis vinifera* [37], respectively. Multifarious functions of different JAZ proteins have been reported previously; for example, in *Arabidopsis*, it was illustrated that AtJAZ proteins could promote growth and reproductive fitness by preventing catastrophic metabolism of an unrestricted immune response [38]. It was also revealed that TaJAZ1 could regulate the expression of ABA-responsive genes and negatively modulate ABA-inhibited seed germination in bread wheat [39]. In rice, OsJAZ1 could act as a transcriptional regulator to interact with OsbHLH148 in the jasmonate signaling pathway, leading to the improvement of drought tolerance [40]. The OsJAZ1-OsCOI1b interaction was characterized to function as a regulator for JA-modulated root and flower development [41]. Additionally, previous studies revealed the Ⅲe transcription factors MYC2, MYC3, MYC4, and MYC5 could function redundantly to regulate JA-mediated stamen development, secondary metabolism, and defenses against insect herbivory and pathogens, and JAZ proteins play a vital role as transcriptional repressors in these biological processes [30,42,43]. Taken together, JAZ proteins have some important effects on regulating growth, defense, and stress responses in plants.

Petunia, as a very popular ornamental plant, occupies a significant portion in the global floriculture industry and plays an essential role as a model plant in genetic research [44,45]. With the diversity of morphology and color, the garden petunia (*Petunia hybrida*) has become the most popular bedding plant all over the world [46]. As we know, crossing two native *Petunia* species is an effective way to obtain garden petunias with superior traits [47]. Therefore, the cultivation of male sterile lines has great significance for the breeding of commercial garden petunias. Genome-wide analysis of gene/protein families has barely been studied in petunia to date, simply owing to the absence of sequence information. Recently, whole-genome sequencing and assembly of two wild petunia parents, *P. axillaris* and *P. inflata*, were successfully conducted to acquire the genome sequences with high quality [46]. The release of the whole-genome sequences of these two *Petunia* progenitors makes it available to conduct genome-wide identification and analysis of gene/protein families. It has been extensively reported that JAZ proteins play critical roles in JA-induced stamen development, leading to male sterility [26,43,48,49]. Here, genome-wide identification and characterization of JAZ family proteins from *P. axillaris* and *P. inflata* genomes were carried out to reveal their sequence properties, phylogenetic relationships, gene structures, motif compositions, and expression profiles. This systematic analysis is expected to lay a foundation for further functional analysis of JAZ proteins with a target of genetic breeding and trait improvement of petunia.

## 2. Materials and Methods

### 2.1. Identification of JAZ Family Proteins in P. axillaris and P. inflata

Two approaches were adopted to identify JAZ family proteins in petunia genomes. Firstly, the whole protein sequences of *P. axillaris* and *P. inflata* were downloaded from Sol Genomics Network (SGN, https://www.sgn.cornell.edu/). Twelve canonical *Arabidopsis* JAZ proteins were download from TAIR (http://www.arabidopsis.org/) and were used as a query to search for JAZ proteins in two petunia protein databases by local blast tool (ftp://ftp.ncbi.nlm.nih.gov/blast/executables/blast+/LATEST) using the Blastp method with an e-value of 1e^−5^. Then, the hidden Markov model (HMM) profiles of the TIFY (PF06200) and the Jas (PF09425) domains were respectively extracted from Pfam databases (http://Pfam.sanger.ac.uk/), and the HMMER 3.0 software (http://hmmer.org/) was used to perform local HMM searches in the two petunia databases aforementioned [50]. All candidate sequences were submitted to domain analysis using InterProScan (http://www.ebi.ac.uk/Tools/pfa/iprscan5/) and SMART (http://smart.embl-heidelberg.de/) tools with default parameters to substantiate the existence of the two conserved domains and determine the exact location of them. Protein sequences without any one domain were rejected.

Additionally, the sequence properties of putative JAZ proteins were analyzed using the online ProtParam tool (http://web.expasy.org/protparam/), including the length of protein sequences (aa), the molecular weight (MW), the theoretical isoelectric point (pI), and the grand average of hydropathicity (GRAVY). Plant-mPLoc (http://www.csbio.sjtu.edu.cn/bioinf/plant-multi/) was employed to predict the subcellular localization of putative JAZ proteins.

### 2.2. Homologous Sequence Alignment and Phylogenetic Analysis

To check out the characteristics of the conserved sequences and the frequency of the most dominant amino acid at each site within the TIFY and the Jas domains, multiple alignment analysis of putative petunia JAZ protein sequences containing these two domains was performed using DNAMAN 7 software with its default settings. The sequence logos of the TIFY and the Jas domains were created by WebLogo 3 (http://weblogo.threeplusone.com/).

Phylogenetic analysis was conducted to determine evolutionary relationships of JAZ family proteins in *Arabidopsis*, rice, tomato, grape, and petunia. MEGA 7.0 software was adopted to construct the phylogenetic tree using the Neighbor Joining (NJ) method with 1000 replicates of bootstrapping to test the reliability [51]. All members of the JAZ family proteins in *A. thaliana* were downloaded from the TAIR database, whereas those proteins in *O. sativa*, *S. lycopersicum*, and *V. vinifera* were acquired from the databases TIGR (http://rice.plantbiology.msu.edu/), SGN, and Phytozome v12.1 (https://phytozome.jgi.doe.gov/), respectively.

### 2.3. Analysis of Conserved Motif and Gene Structure

A phylogenetic tree was also generated using only the 28 JAZ proteins to study the structural features based on their evolutionary relationships in petunia. The peptide sequences of the JAZ proteins were submitted to the online MEME 5.0.4 (http://meme-suite.org/tools/meme) for the analysis of conserved motif composition; 10 motifs were set as the maximum number to find with the width between 6 and 120 amino acids, while other settings were at default values.

The coding sequences and the corresponding genomic sequences of each petunia *JAZ* gene were submitted to Gene Structure Display Server (GSGD, http://gsds1.cbi.pku.edu.cn/) for the analysis of gene structure, including sequence length, exon/intron organization, and upstream/downstream regions.

### 2.4. Analysis of Cis-Acting Regulatory Elements

Twelve *PaJAZ* genes were selected to analyze the putative *cis*-acting regulatory elements in a promoter region, and the PlantCARE (http://bioinformatics.psb.ugent.be/webtools/plantcare/html/) was employed to carry out the analysis using 1.5 kb sequences upstream of the start codon in each *PaJAZ* gene.

### 2.5. Calculation of Ka/Ks Ratios

In genetics, the Ka/Ks value represents the ratio between the non-synonymous substitution rate (Ka) and the synonymous substitution rate (Ks) of two protein-coding genes. The ratio can be adopted to determine whether there is selective pressure on the protein-coding genes [52]. In this study, several paralogous pairs of petunia *JAZ* genes were aligned using MEGA 7.0 software. Then, the Ka, the Ks, and the Ka/Ks ratios were estimated by DnaSP v5 [53]. Divergence time (T) was evaluated by T = Ks/(2 × 9.1 × 10^−9^) × 10^−6^ million years ago (Mya) [54].

### 2.6. Plant Materials, MeJA Treatment, RNA Isolation, and Gene Expression Analysis

*P. hybrida* ‘Fantasy Red’ was used for gene cloning and expression analyses, which were planted at the experimental base of Huazhong Agricultural University, Wuhan, China. The seven different developmental stages of flower buds (0.2b, 0.3b, 0.5b, 1.0b, 1.5b, 2.5b, and 3.5b) and anthers (0.2a, 0.3a, 0.5a, 1.0a, 1.5a, 2.5a, and 3.5a) were sampled on the basis of flower bud length at 0.2, 0.3, 0.5, 1.0, 1.5, 2.5 and 3.5 cm (deviation range ±0.2 mm) without sepals (Figure S1), and the plant tissues of young roots, stems, leaves, and opening flowers were separately collected from three individual plants. In addition, *P. hybrida* ‘Mitchell’ was used for MeJA treatment and further expression analyses. Three individual plants were sprayed with 100 μM MeJA, and the control plants were treated with ddH_2_O. Samples of flowers buds and anthers at the seven developmental stages were also separately collected after 8 h for treatment. All samples were instantly frozen in liquid nitrogen and stored at −80 °C for further analysis.

Total RNA was isolated from different samples using RNAiso Reagent (TaKaRa, Japan) on the basis of our previous method [55,56]. First-strand cDNA synthesis was executed using a TransScript One-Step gDNA Removal and cDNA Synthesis SuperMix kit (Transgene, Beijing, China) following the manufacturer’s instruction [57]. The qRT-PCR was performed using SYBR^®^ Premix Ex Taq^TM^ kit (Takara, Dalian, China) on the ABI 7500/7500-Fast Real-Time PCR System (Applied Biosystems, Foster City, CA, USA) according to a previously described method [58]. The 2^−^^△△Ct^ method was used to evaluate the expression level. Three independent replications were set for each experiment, and data were presented as mean values ± standard error. The housekeeping *β-actin* gene was used as the internal control. All primers were designed by Primer 5.0 software and listed in Table S2.

### 2.7. Subcellular Localization Assays

Three pairs of gene-specific primers (listed in Table S2) were designed to amplify the coding sequences of *PaJAZ5*, *PaJAZ9*, and *PaJAZ12* without the stop codon from cDNA temples, and the PCR products of these genes were separately inserted into the *FRET-YFP* vector. An empty vector and the recombinant constructs *35s::PaJAZs::YFP* were transformed into the competent cell of *Agrobacterium tumefaciens* strain GV1301. Then, the transformed *Agrobacterium* strain was incubated and further infiltrated into tobacco (*Nicotiana benthamiana*) leaves for transient expression. A confocal laser scanning microscope (Leica TCS SP8, Germany) was employed to observe the localization of the fluorescence after infiltration for 48 h.

### 2.8. Y2H Assays

The coding sequences of *PaJAZ1-12*, *MYC2*, and truncated derivatives (*MYC2^NT30^*^0^, *MYC2^CT350^*, *MYC2^NT88^*, *MYC2^CT562^*) were cloned with gene-specific primers (listed in Table S2). The PCR products of *PaJAZ1-12* were inserted into pGADT7 to construct prey vectors, and *MYC2* and truncated derivatives were inserted into pGBKT7 to construct bait vectors, respectively. Then, the recombinant constructs were confirmed by sequencing.

To test the autoactivation of the bait vectors, they were respectively transformed into yeast (*Saccharomyces cerevisiae*) strain AH109. The transformed yeast cells were confirmed by normal growth on solid synthetic dropout (SD) medium lacking Trp (SD/-T). Three days later, transformed yeast cells were incubated in selective SD/-T liquid medium for 6 h and then suspended to an OD_600_ of 0.8. Samples (3 μL) of a series of cell suspensions with different concentration gradients (10x dilutions) were plated on solid SD medium lacking Trp and Ade (SD/-TA) to test the autoactivation after 30 °C incubation for 3 d.

To assess protein–protein interactions, the corresponding bait and prey vectors were co-transformed into AH109. Successfully transformed strains were identified on SD medium lacking Trp and Leu (SD/-TL). To test the interactions, transformed strains were grown in liquid SD/-TL medium and then suspended to an OD_600_ of 0.8. Samples (3 μL) of different cell suspensions were plated out on solid SD medium lacking Trp, Leu, His, and Ade (SD/-TLHA) to detect the interactions after 30 °C incubation for 3 d.

## 3. Results

### 3.1. Identification of JAZ Family Proteins in P. axillaris and P. inflata

HMM and Blastp searches were conducted to identify petunia JAZ proteins. In total, 12 and 16 JAZ proteins were identified in *P. axillaris* and *P. inflata* genomes. Then, they were respectively designated as PaJAZ1-12 and PiJAZ1-16 according to the naming convention (Table 1). Furthermore, in order to corroborate the reliability of the initial results, the online InterProScan and SMART tools were used to check the existence of the conserved domains with the default parameters. The results revealed that all of the 28 putative JAZ proteins contained highly conserved TIFY and Jas domains. The exact locations of these two domains in different JAZ protein sequences are listed in Table 1.

Bioinformatics analyses showed that the length of petunia JAZ proteins ranged from 96 to 393 amino acids (aa) with an average of 234 aa, and the molecular weight varied from 10.85 to 41.65 kDa with a pI between 5.94 and 10.39. Inferred from the pI features, all proteins except PaJAZ10 and PiJAZ14 were basic proteins (pI > 7), and the GRAVY indicated that all these proteins were hydrophilic, since the values were negative. Moreover, all of these JAZ proteins were predicted to localize to the nucleus with the use of Plant-mPLoc. The results provide a basis for better characterization of JAZ family proteins in petunia.

### 3.2. Homologous Sequences Alignment and Phylogenetic Analysis

Multiple alignment analysis was conducted using the homologous sequences of putative petunia JAZ proteins containing TIFY and Jas domains, and it suggested that these two domains were highly conserved in all petunia JAZ proteins in spite of their difference in sequence lengths and properties (Figure S2). It has been reported that the TIFY domain could mediate hetero- and homomeric interactions among JAZ proteins, and the Jas domain is associated with extensive protein–protein interactions. The high conservation of these two domains is indicative of their functional similarity in petunia. From the alignment, we can also see that some JAZ proteins, including PaJAZ3, PaJAZ12, PiJAZ7, PiJAZ8, and PiJAZ16, possess a nonclassical “TMFY” motif instead of the “TIFY” motif that is prevalent in other plants.

**Figure 1:**
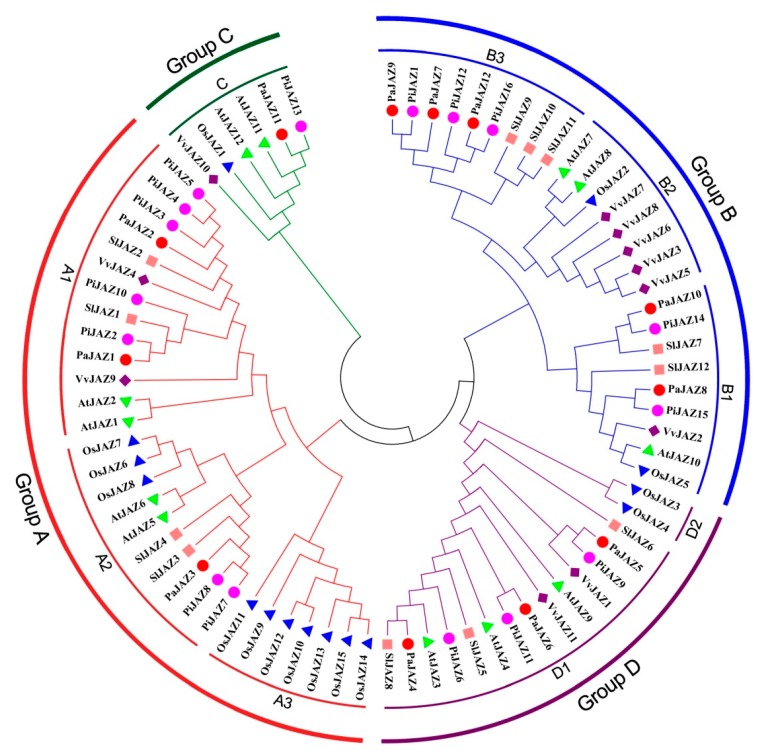
Phylogenetic tree for JAZ protein family from petunia, *Arabidopsis*, grape, rice, and tomato. MEGA 7.0 was adopted for phylogenetic analysis by using the Neighbor Joining (NJ) method with 1000 replicates of bootstrapping to test the reliability.

To discover the evolutionary relationships of JAZ family proteins between the two *Petunia* progenitors and *A. thaliana*, *O. sativa*, *S. lycopersicum*, and *V. vinifera*, a phylogenetic tree was generated by the NJ method based on multiple sequences alignment of 12 AtJAZ proteins, 15 OsJAZ proteins, 15 SlJAZ proteins, 11 VvJAZ proteins, 12 PaJAZ proteins, and 16 PiJAZ proteins. As shown in Figure 1, according to the phylogenetic grouping of JAZ proteins in *Arabidopsis* [27], all 78 JAZ protein members obtained from different plants could be divided into four groups (Groups A–D) and further classified into eight subgroups (A1–3, B1–3, C, and D1–2). All petunia JAZ proteins were distributed into six subgroups (A1, A2, B1, B3, C, and D1), while none were presented in the rest of subgroups. In general, the phylogenetic tree revealed that there was a closer relationship between petunia and tomato rather than *Arabidopsis*, grape, and rice in terms of the JAZ protein family, which tallied with the current established evolutionary relationships in plants [59].

### 3.3. Conserved Motif and Gene Structure Analysis

**Figure 2:**
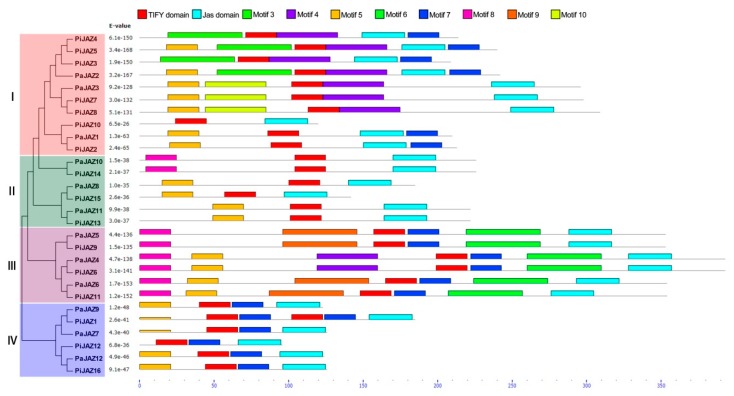
Analysis of motif composition in petunia JAZ proteins in the light of phylogenetic relationship. MEME database was used for motif analysis with the complete amino acid sequences of petunia JAZ proteins.

As shown in Figure 2, petunia JAZ proteins were classified into four distinct clusters (I, II, III, and IV) with a similar topology of the phylogenetic tree constructed above. MEME database search identified 10 conserved motifs (Figure S3); Motifs 1 and 2 were identified as the TIFY domain and the Jas domain, respectively, but the others had no functional annotations. Although the lengths of petunia JAZ protein sequences were discrepant, members that clustered together tended to possess a significantly similar number and composition of conserved motifs. On the other hand, each cluster shared similar motif features, which could further support the phylogenetic grouping of the JAZ family.

**Figure 3:**
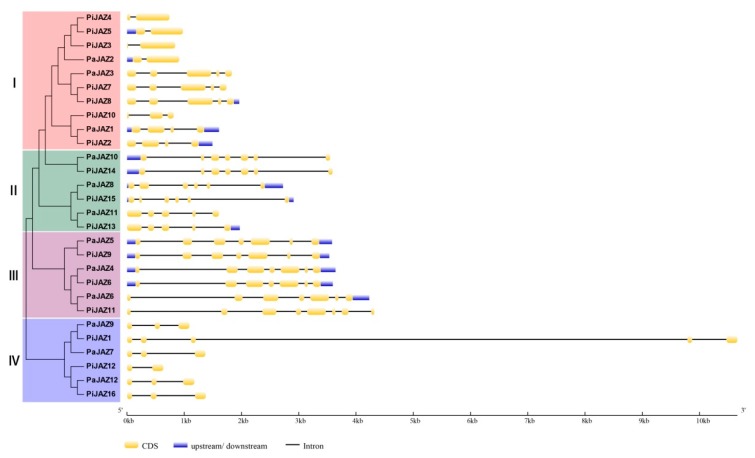
Analysis of gene structure in petunia *JAZ* genes in the light of the phylogenetic relationship. GSDS database was adopted for exon/intron distribution analysis. Yellow boxes, blue boxes, and black lines indicate exons, upstream/downstream, and introns, respectively.

The divergence in the gene structure could promote the evolution of gene families to support the phylogenetic classification [60]. Among the petunia JAZ family genes, the number of introns ranged from one to seven, and exons ranged from one to eight. The results implied that a strong correlation existed between gene structure and phylogenesis (Figure 3). Petunia *JAZ* genes that clustered at the branch tips of the phylogenetic tree usually possessed a similar gene structure—that is, members in one cluster generally showed similar exon/intron numbers and distribution patterns. For instance, the orthologous pairs *PaJAZ10*/*PiJAZ14*, *PaJAZ5*/*PiJAZ9*, or *PaJAZ4*/*PiJAZ6* had almost the same gene structures, and they displayed a closer evolutionary relationship. Moreover, we also noted that all JAZ genes in cluster IV had no upstream/downstream regions.

### 3.4. Analysis of Cis-Acting Regulatory Elements

*Cis*-acting regulatory elements function as molecular switches tightly associated with the regulation of gene expression under biotic and abiotic stresses [61]. The analysis of *cis*-elements could provide potential evidence for further functional analysis of the petunia JAZ family proteins. Therefore, a variety of stress-responsive elements was detected in the putative promoter regions of each *PaJAZ* gene using PlantCARE database (Table S1), including ABA-responsive elements (ABRE), SA-responsive elements (TCA-element), ethylene-responsive elements (ERE), elements involved in the low-temperature response (LTR), and elements involved in defense and stress responsiveness (TC-rich repeats). In particular, *cis*-acting elements involved in JA-mediated responses, including the MeJA-responsive elements (TGACG-motif and CGTCA-motif), as well as the wound-responsive elements (WUN-motif) and the MYC-binding site (G-box) were also discovered. The result revealed that these *PaJAZ* genes were likely to be involved in the responses to related biotic or abiotic stresses.

### 3.5. Calculation of Ka/Ks Ratios

Ka/Ks ratio can be calculated to evaluate whether there is selective pressure behaving on the protein-coding genes with positive selection (Ka/Ks ratio >1), neutral selection (ratio = 1), and purifying selection (ratio < 1) [52]. The analysis demonstrated that the Ka/Ks ratios of the seven petunia *JAZ* paralogous pairs were less than one (ranged from 0.13 to 0.51), suggesting that these *JAZ* genes had mainly undergone purifying selection in the evolutionary processes (Table 2). According to the formula of calculating divergence time, the seven paralogous pairs were estimated to have diverged between 2.14 Mya and 39.61 Mya with a mean of 23.08 Mya.

### 3.6. The Expression Analysis of PaJAZ Genes

**Figure 4:**
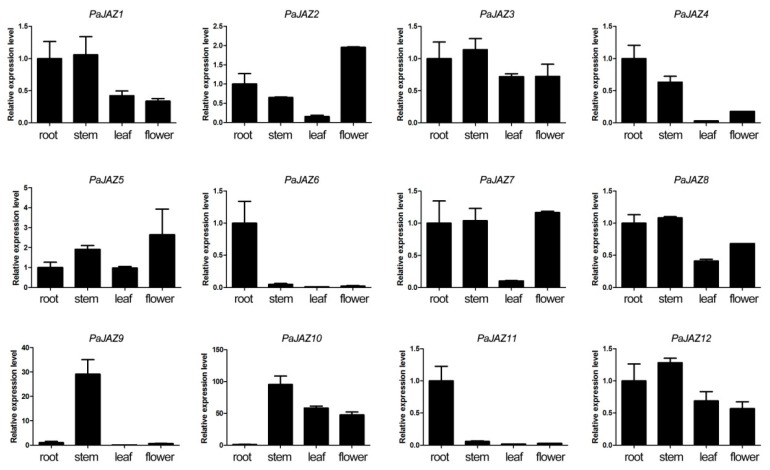
The expression patterns of *PaJAZ* genes in different petunia tissues. The RNA samples of roots, stems, leaves, and flowers were extracted from *P. hybrida* ‘Fantasy Red’. The expression levels of *PaJAZ* genes were detected by the 2^−^^△△Ct^ method with petunia *β-actin* gene as the internal control, and the expression in the root was defined as one. The data were calculated from three replications and presented as mean values ± standard error.

To assess the expression levels of the *PaJAZ* genes in specific tissues, petunia roots, stems, leaves, and flowers were used for RNA isolation and further qRT-PCR analyses. The results are shown in Figure 4. The expression profiles of *PaJAZ* genes in these four tissues were diverse but could be generally divided into three types. Some genes were constitutively expressed in these four tissues, namely, *PaJAZ1*, *3*, *5*, *8*, and *12*; some genes, including *PaJAZ6*, *9*, and *11*, had specific expression characteristics to some extent, and others were weakly expressed in a particular tissue. For instance, *PaJAZ4* and *PaJAZ7* were weakly expressed in leaves compared to the other tissues, and *PaJAZ10* had a relatively lower expression level in roots.

**Figure 5:**
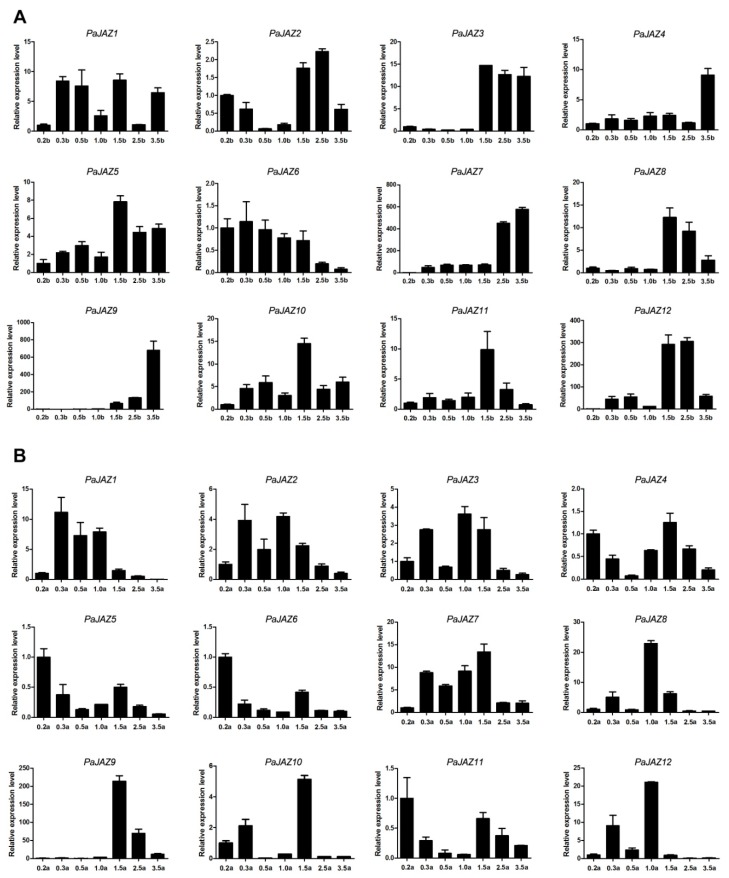
The expression analysis of *PaJAZ* genes during the development of petunia flower buds (**A**) and anthers (**B**). The RNA samples isolated from flower buds and anthers at seven developmental stages in *P. hybrida* ‘Fantasy Red’. The expression levels of *PaJAZ* genes were detected by the 2^−^^△△Ct^ method with petunia *β-actin* gene as the internal control, and the expression in 0.2b (0.2a) stage was defined as one. The data were calculated from three replications and presented as mean values ± standard error.

In order to further study the expression patterns of *PaJAZ* genes during the developmental processes of flower buds and anthers in petunia, flower buds of different lengths representing different developmental stages were used for RNA isolation and gene expression analysis. From the perspective of flower buds at different developmental stages (Figure 5A), except for *PaJAZ1* and *PaJAZ6*, the expressions of the rest of the *PaJAZ* genes were at a lower level in the early developmental stages of flower buds but increased later. On the contrary, the expression of *PaJAZ6* gradually decreased with the development of flower buds, but the expression pattern of *PaJAZ1* was irregular. Interestingly, the expression of several genes, including *PaJAZ3*, *4*, *7*, *8*, *9*, and *12*, changed dramatically during two developmental stages. For example, the expression of *PaJAZ3* increased sharply from 1.0b to 1.5b stages and remained at a high level to the last developmental stage, implying that these *PaJAZ* genes might have played significant roles in such periods.

During the development of petunia anthers, the expressions of *PaJAZ* genes were more fluctuating, but there were certain rules to some degree (Figure 5B). Except for *PaJAZ4*, *5*, *6*, *9*, and *11*, the expression of the remaining genes generally increased at 0.3a and 1.0a stages of anther development, and the corresponding expression patterns showed some similarities. *PaJAZ4*, *5*, *6*, and *11* had a relatively high expression level at 0.2a and 1.5a stages, exhibiting almost the same expression patterns. The majority of *PaJAZ* genes had a lower expression level at 3.5a stage compared with that in 0.2a stage with the exception of *PaJAZ7* and *PaJAZ9*. It was noteworthy that the expression of *PaJAZ9* increased remarkably from 1.5a to 2.5a, indicating that it might play a critical role in this period. In addition, *PaJAZ1*, *2*, and *3* (or *PaJAZ4*, *5*, and *6*) displayed similar expression patterns, which were clustered into one cluster in phylogenetic tree, suggesting their functional consistency during anther development.

**Figure 6:**
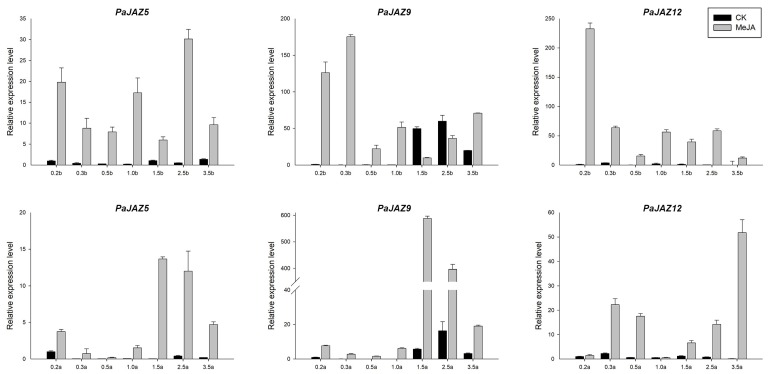
The expression analysis of *PaJAZ5*, *PaJAZ9*, and *PaJAZ12* genes under MeJA treatment. The RNA samples isolated from flower buds and anthers at seven developmental stages in control and MeJA treated plants of *P. hybrida* ‘Mitchell’. The expression levels of *PaJAZ* genes were detected by the 2^−^^△△Ct^ method with petunia *β-actin* gene as the internal control, and the expression in control plants at 0.2b (0.2a) stage was defined as one. The data were calculated from three replications and presented as mean values ± standard error.

To reveal the expression patterns under MeJA treatment, *PaJAZ5*, *PaJAZ9*, and *PaJAZ12* genes were selected to conduct qRT-PCR analyses. As shown in Figure 6, except *PaJAZ9* at 1.5b and 2.5b stages, the expression levels of these three genes were generally up-regulated during the development of flower buds and anthers after MeJA treatment, and the changes were extremely significant at some stages. Furthermore, the expression patterns of these three genes after the treatment were fluctuating in flower buds, but it showed relatively high expression levels at the later developmental stages in anthers.

### 3.7. Subcellular Localization of PaJAZ Proteins

**Figure 7:**
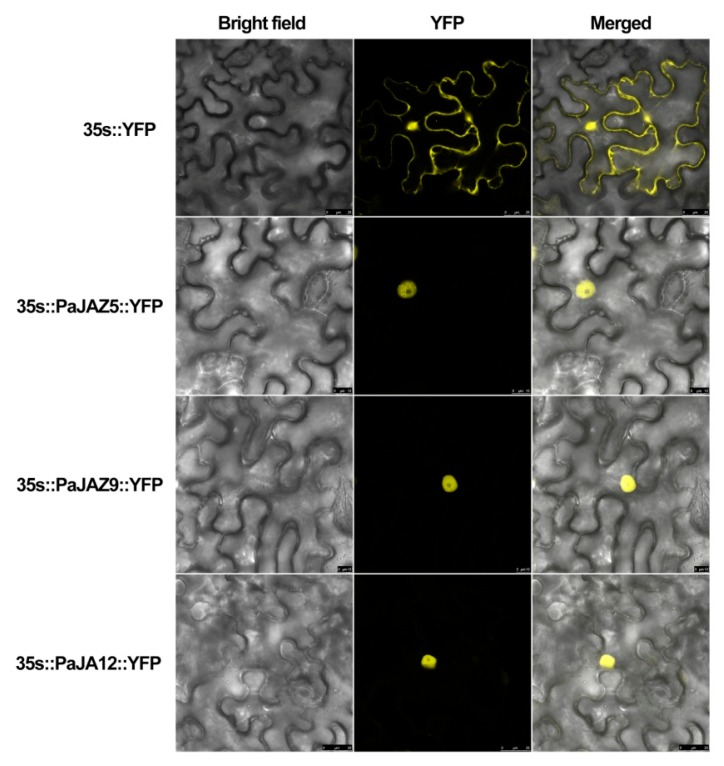
Subcellular localization assays of PaJA5, PaJAZ9, and PaJAZ12 proteins in tobacco leaf cells; 35s::YFP was used as the positive control.

Due to their differential expression patterns, *PaJAZ5*, *PaJAZ9*, and *PaJAZ12* were selected to construct the *35s::PaJAZs::YFP* fusion vectors and then transiently expressed in leaves of tobacco to investigate the subcellular localization. A Leica confocal laser scanning microscopy was adopted to observe the fluorescence in leaf epidermal cells. As shown in Figure 7, YFP fluorescence of the 35s::YFP could be detected in the whole leaf cells, and the fluorescence of three fusion proteins was mainly accumulated in cell nuclei, indicating that PaJAZ5, PaJAZ9, and PaJAZ12 proteins localized in the cell nucleus. The results were consistent with the predication of subcellular localization using Plant-mPLoc.

### 3.8. Interacion Between PaJAZ Proteins and Transcription Factor MYC2

**Figure 8:**
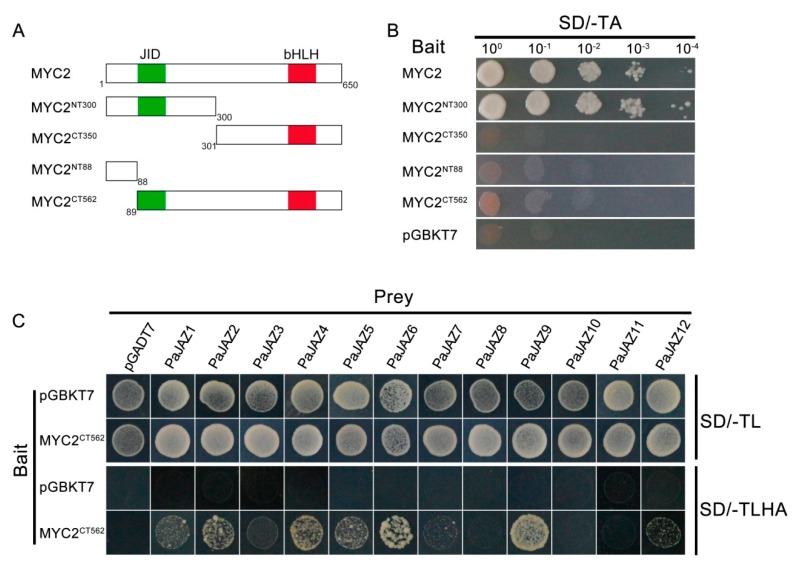
(**A**) Schematic diagrams showed the truncated derivatives of MYC2. The diagrams displayed the conserved JAZ-interaction (JID) domain (green) and basic helix-loop-helix (bHLH) domain (red). The first and the last amino acids of the truncated derivatives are presented in numbers below. (**B**) The autoactivation test of full-length MYC2 or several truncated derivatives. Yeast cell AH109 carrying a recombinant bait vector was plated on solid synthetic dropout medium lacking Trp (SD/-TA) to test autoactivation. (**C**) Y2H assays to test the interaction between PaJAZ proteins and transcription factor MYC2. A combination of recombinant pGBKT7-MYC2^CT562^ (bait) and various pGADT7-PaJAZ constructs (prey) was co-transformed into yeast strain AH109 and further plated on SD medium lacking Trp and Leu (SD/-TL) as a control or SD medium lacking Trp, Leu, His, and Ade (SD/-TLHA) to test protein interactions.

As a first step for any Y2H assays, it is essential to verity that the bait will not automatically activate reporter genes without a prey protein. At this point, full-length MYC2 or several MYC2 truncated derivatives were fused to pGBKT7 vectors to construct bait vectors and then transformed into yeast competent cells AH109 to test the autoactivation. As shown in Figure 8A,B, the cells transformed the bait vectors fused full-length MYC2 or derivative MYC2^NT300^ exhibited autoactivation of growing on the SD/-TA medium. However, derivatives MYC2^CT350^, MYC2^NT88^, and MYC2^CT562^ did not exhibit autoactivation.

The transcription factor MYC2 contained an N-terminus JAZ-interaction (JID) domain, which was characterized to mediate the interaction with JAZ repressors and the basic helix-loop-helix (bHLH) domain in C-terminus required for homo- or hetero-dimerization [14]. In *Arabidopsis*, AtMYC2 could interact with practically all JAZ family proteins with the exception of AtJAZ4 and AtJAZ7 [27]. We did firstly plan to test the protein–protein interactions using MYC2-prey and PaJAZ-bait as the strategy in *Arabidopsis*, but it seemed that MYC2-prey was toxic to the yeast cells that could not grow normally. Given this, we constructed several bait vectors of MYC2 truncated derivatives and selected the MYC2^CT562^ containing the JID domain but exhibiting no autoactivation to check the capacity of MYC2 to interact with all JAZ family proteins in *P. axillaris.* The interaction between petunia MYC2 and JAZ proteins was assessed using a Y2H assay. As shown in Figure 8C and Figure S4, most PaJAZ proteins could interact with MYC2 in yeast. Furthermore, a relatively strong interaction was observed between MYC2 and PaJAZ2, 4, 5, 6, and 9, and a relatively weak interaction was detected with PaJAZ1, 3, 7, and 12. However, PaJAZ8, 10 and 11 could not interact with transcription factor MYC2 in Y2H assays.

## 4. Discussion

JA is widely involved in the regulation of growth, development, and defense in plants [5,62]. The plant-specific JAZ family proteins act as negative regulators of JA-responsive genes [9]. In recent years, JAZ protein families have been identified in some important agricultural and economical crops, such as rice [33], maize [63], wheat [34], cotton [64], tomato [36], strawberry [65], sugarcane [66], and grape [37]. However, the evolutionary and the expression analyses of the JAZ protein family in petunia have not been reported to date. Based on the released genome data, the present study aimed to conduct a comprehensive identification of the JAZ protein family in petunia and to systematically analyze their sequence characteristics, evolutionary relationships, subcellular locations, and expression profiles.

A total of 12 and 16 JAZ proteins were respectively identified in *P. axillaris* and *P. inflata* genomes. It was likely that the JAZ family genes in *P. inflata* might have experienced more times of gene duplication events, which led to more members in the genome, thereby resulting in the absence of the orthologous pairs for some genes in *P. axillaris*. Multiple sequence alignments revealed that all of these proteins possessed a conserved TIFY domain and a Jas domain. It has been reported that the “TIFY” motif has several deviations in other plants: VIF[F/Y]XG, TLF[F/Y]XG, TLL[F/Y]XG, TLS[F/Y]XG, TLV[F/Y]XG, TMF[F/Y]XG, TII[F/Y]XG, and TIS[F/Y]XG [23]. Here, we found some slight deviation also existed in the core “TIFY” motif in petunia; the core sequence of “TIFY” was replaced with “TMFY” in PaJAZ3, PaJAZ12, PiJAZ7, PiJAZ8, and PiJAZ16. This result illustrated the structural diversity of the core “TIFY” motif, which indicated that they might have different functions. Conversely, the sequences of the Jas domain were relatively conserved, which played an important role in regulating the perception of JA sensitivity and the interaction with transcription factors [9,10,15]. Additionally, the Jas domain was verified to be involved in subcellular localizations of JAZ proteins [67].

In line with the NJ phylogenetic tree, the JAZ family proteins derived from different plants could be divided into four groups (Group A, B, C, and D) and further classified into eight subgroups (A1–3, B1–3, C, D1–2). The petunia JAZ family proteins were distributed in subgroups A1, A2, B1, B3, C, and D1, while none were presented in the rest of the subgroups. Interestingly, subgroups A3 and D2 were entirely composed of rice JAZ proteins, implying the distant relationship between monocots and dicots, and it was clear that there was a closer relationship between petunia and tomato. Moreover, the members in different clusters of the petunia JAZ family presented significant divergence in exon/intron organization, sequence length, and motif composition, but those in one cluster were tightly similar in these characteristics. In addition, the divergence in the motif composition and the gene structure could provide additional evidence to support phylogenetic groupings [68]. In terms of gene structure, the multiformity is predominantly reflected in the number and the length of introns. In this study, all the petunia JAZ family genes contained at least one intron, even though the number and the lengths of introns were discrepant, and the *JAZ* genes lacking introns was only found in rice, wheat, and maize [33,34,63], which might reflect the evolutionary differences between monocots and dicots. The TIFY and the Jas domains are necessary for JAZ proteins to function as transcription repressors. As shown in Figure 2, each petunia JAZ protein contained an N-terminus TIFY domain and a C-terminus Jas domain, which suggested the high degree of conservation in structures. Moreover, protein members in one cluster also displayed a similar motif composition. Additionally, the Ka/Ks ratio was calculated to assess the driving force underlying the evolution of petunia *JAZ* genes. The result indicated that the petunia JAZ family genes mainly experienced purifying selection—that is, they had the tendency to be stable during the evolutionary process. Unfortunately, due to the limitation of genomic data, chromosome locations, duplication events, and microsynteny analyses could not be conducted, which need to be accomplished after the genome is assembled to the chromosome level.

*Cis*-acting regulatory elements play a critical role in regulating the expression of related genes by controlling promoter efficiency [69]. In this research, the *cis*-acting elements related to phytohormones (MeJA, ABA, ethylene, and SA) and stress responses (low temperature and drought) were discovered in the putative promoter regions of different petunia *JAZ* genes. This suggested that the expression levels of a few *JAZ* genes were likely to be up- or down-regulated under such biotic or abiotic stresses. Previous research demonstrated a negative feedback loop that was generated by promoting the transcription repressors to express and then attenuate the JA signal. JAZ proteins bind to transcription factors to repress various JA-mediated responses, and the ubiquitination and the degradation of JAZ proteins result in the release of JAZ-interacting transcription factors, thereby facilitating the expression of early JA-responsive genes, including related transcription factors and some *JAZ* genes themselves [9,29,70,71,72]. Here, the Y2H assays elucidated that most PaJAZ proteins could interact with transcription factor MYC2, and it was noteworthy that most petunia *JAZ* genes (*PaJAZ 2–5*, *8–10*, *12*) had at least one G-box, the target binding site of transcription factor MYC2 [13,73], in the promoter regions, implying the existence of the negative feedback loop associated with JAZ proteins and transcription factor MYC2 in petunia.

The tissue-specific expression analysis was carried out to assess the expression levels of *PaJAZ* genes in different tissues of petunia. In general, the expression patterns of *PaJAZ* genes in different tissues were discrepant; some genes showed constitutive expression patterns in four tissues (*PaJAZ1*, *3*, *5*, *8*, and *12*), some were specifically expressed in specific tissues (*PaJAZ6*, *9*, and *11*), and others had a relatively lower expression levels in a particular tissue (*PaJAZ4*, *7*, and *10*). It suggested that different *PaJAZ* genes might participate in the regulation of growth and development of different tissues in petunia. Except *PaJAZ1* and *PaJAZ6*, the expressions of the rest of *PaJAZ* genes were at a higher level in the late stages, when these genes might be involved in regulating the development of flower bud. The expression of *PaJAZ* genes in petunia anthers was fluctuant, but some displayed time-specific characteristics, such as *PaJAZ8*, *9*, *10*, and *12*. It was noteworthy that the expression levels of some *PaJAZ* genes, including *PaJAZ2*, *3*, *5*, *8*, *10*, *11*, and *12*, changed sharply from 1.0b to 1.5b stages, which was likely to be the transition period for flower bud maturity. Contrary to the expression pattern in flower buds, almost all *PaJAZ* genes had a respectively lower expression level at the late stages of anther development but highly expressed in earlier stages. Additionally, *PaJAZ5*, *9*, and *12* were selected to perform the expression analyses under MeJA treatment, which displayed generally higher expression levels after the treatment, indicating the expression of these *PaJAZ* genes were induced by JA. This further provided more potential evidence for the negative feedback loop in the JA signaling pathway. In *Arabidopsis*, JA has been reported to be essential for stamen development [43,74,75,76,77,78], and the JAZ proteins are the key repressors in the JA signaling pathway, thus the expression levels of *PaJAZ* genes at different stages are most likely to be involved in the changes of the endogenous JA level during the development of petunia. Given this, more functional analyses will be imperatively adopted to reveal the underlying molecular mechanisms in JA signaling. Furthermore, some genetic engineering technologies, such as CRISPR/Cas9 and RNA interference, are expected to be used in the breeding of petunia male sterile lines on the basis of some functional *JAZ* genes.

## 5. Conclusions

In summary, a total of 28 JAZ family proteins were identified from two petunia progenitors, *P. axillaris* and *P. inflata*. In line with the phylogenetic tree, the 28 proteins could be divided into four groups (Group A–D) and further classified into six subgroups based on gene structure and motif composition. The calculation of Ka/Ks ratio revealed that petunia JAZ family genes had been influenced principally by the purifying selection. *Cis*-acting regulatory elements analysis laid a foundation for further research on the function of JAZ family genes in responses to related biotic or abiotic stresses. The Y2H assays demonstrated nearly all the PaJAZ proteins could interact with transcription factor MYC2. The qRT-PCR analysis suggested that different *PaJAZ* genes displayed different expression patterns, and it could be inferred that PaJAZ proteins may play key roles in various aspects of plant growth, including anther development. Overall, this research could provide the foundation for further functional analysis of JAZ proteins and the biotechnological resources for genetic engineering breeding in petunia.

## Figures and Tables

**Table 1:** The sequence properties of the 28 JAZ family proteins identified in *P. axillaris* and *P. inflata* genome.

Name	Protein ID in SGN ^1^	Length ^2^	MW ^3^	pI	GRAVY ^4^	TIFY ^5^	Jas ^6^
PaJAZ1	Peaxi162Scf00533g00059.1	210	23.09	9.36	−0.485	84–115	152–176
PaJAZ2	Peaxi162Scf00928g00130.1	242	26.98	9.38	−0.596	101–134	180–204
PaJAZ3	Peaxi162Scf00038g01961.1	296	32.35	9.31	−0.685	99–132	240–264
PaJAZ4	Peaxi162Scf00314g00330.1	393	41.59	9.36	−0.302	196–229	332–356
PaJAZ5	Peaxi162Scf00376g00327.1	353	37.57	9.15	−0.229	154–187	292–316
PaJAZ6	Peaxi162Scf00207g00008.1	354	37.45	9.83	−0.364	164–195	297–321
PaJAZ7	Peaxi162Scf00317g00117.1	126	14.30	9.62	−0.731	42–74	104–124
PaJAZ8	Peaxi162Scf00128g00175.1	185	20.58	9.46	−0.595	98–128	144–167
PaJAZ9	Peaxi162Scf00317g00121.1	123	13.99	10.02	−0.826	38–69	100–120
PaJAZ10	Peaxi162Scf00305g00018.1	226	25.74	5.94	−0.767	101–134	175–198
PaJAZ11	Peaxi162Scf00011g01124.1	222	24.90	9.02	−0.677	98–130	168–192
PaJAZ12	Peaxi162Scf00394g00120.1	124	14.05	9.67	−0.602	36–68	102–122
PiJAZ1	Peinf101Scf01317g09018.1	185	20.81	9.61	−0.744	42–74,100–131	162–182
PiJAZ2	Peinf101Scf00193g17008.1	213	23.43	9.38	−0.494	86–117	154–178
PiJAZ3	Peinf101Scf03815g00017.1	209	23.56	9.23	−0.512	63–96	148–172
PiJAZ4	Peinf101Ctg13655775g00003.1	214	23.99	9.32	−0.602	68–101	153–177
PiJAZ5	Peinf101Scf03816g00028.1	240	26.79	9.24	−0.595	101–134	180–204
PiJAZ6	Peinf101Scf01633g04032.1	393	41.65	9.2	−0.284	196–229	332–356
PiJAZ7	Peinf101Scf06002g00004.1	298	32.58	9.33	−0.758	99–132	242–26
PiJAZ8	Peinf101Scf03176g00010.1	309	33.77	9.22	−0.745	110–143	253–277
PiJAZ9	Peinf101Scf00497g02012.1	353	37.62	8.84	−0.238	154–187	292–316
PiJAZ10	Peinf101Scf00048g00001.1	120	13.39	9.52	−0.592	22–53	88–103
PiJAZ11	Peinf101Scf01889g01055.1	354	37.47	9.69	−0.395	147–178	280–304
PiJAZ12	Peinf101Scf00637g07016.1	96	10.85	10.39	−0.822	8–40	74–94
PiJAZ13	Peinf101Scf02714g01041.1	222	24.80	9.21	−0.724	98–130	168–192
PiJAZ14	Peinf101Scf00947g00007.1	226	25.85	6.01	−0.735	101–134	175–198
PiJAZ15	Peinf101Scf00889g01051.1	142	16.22	9.44	−0.679	55–85	101–124
PiJAZ16	Peinf101Scf00280g05010.1	126	14.44	9.81	−0.66	41–73	104–124

^1^ SGN: Sol Genomics Network; ^2^ the length of amino acid sequences; ^3^ the molecular weight of proteins (kDa); ^4^ the grand average of hydropathicity; ^5, 6^ the location of TIFY and Jas domains within the amino acid sequences.

**Table 2:** Ks, Ka, and Ka/Ks ratios calculated for paralogous pairs of petunia *JAZ* genes.

Paralogous pairs	Ka	Ks	Ka/Ks Ratio	Date (Mya)
*PaJAZ4-PaJAZ6*	0.0082	0.0626	0.13	3.44
*PaJAZ7-PaJAZ9*	0.0092	0.0390	0.24	2.14
*PiJAZ2-PiJAZ10*	0.1406	0.4864	0.29	26.73
*PiJAZ4-PiJAZ5*	0.1792	0.5381	0.33	29.57
*PiJAZ6-PiJAZ11*	0.3269	0.7209	0.45	39.61
*PiJAZ7-PiJAZ8*	0.3187	0.6282	0.51	34.52
*PiJAZ12-PiJAZ16*	0.2327	0.4651	0.50	25.55

## Supplementary Materials

The following are available online at https://www.mdpi.com/2223-7747/8/7/203/s1. Figure S1: The seven developmental stages of flower buds (0.2b, 0.3b, 0.5b, 1.0b, 1.5b, 2.5b, and 3.5b) and anthers (0.2a, 0.3a, 0.5a, 1.0a, 1.5a, 2.5a, and 3.5a) based on flower bud length at 0.2, 0.3, 0.5, 1.0, 1.5, 2.5, and 3.5 cm (deviation range ±0.2 mm) in *Petunia hybrida* ‘Fantasy Red’. Figure S2: The homologous sequence alignment of petunia JAZ family proteins. Figure S3: The sequence logos of the 10 motifs generated by MEME software. Figure S4: Y2H assays to test the interaction between PaJAZ proteins and transcription factor MYC2. Table S1: The number and composition of *cis*-acting regulatory elements is in the promoter regions of *JAZ* genes in *P. axillaris*. Table S2: The primers used in this study.
