# Supplementary material for: Genome-Wide Identification and Characterization of JAZ Protein Family in Two Petunia Progenitors
Source: Plants (Basel). 2019 Jul 3;8(7):203. doi: 10.3390/plants8070203 (PMC6681285; doi:10.3390/plants8070203)
Supplement: Supplementary file 1 [file plants-08-00203-s001.zip › Supplementary Materials-proofreading/Figure S1.docx]

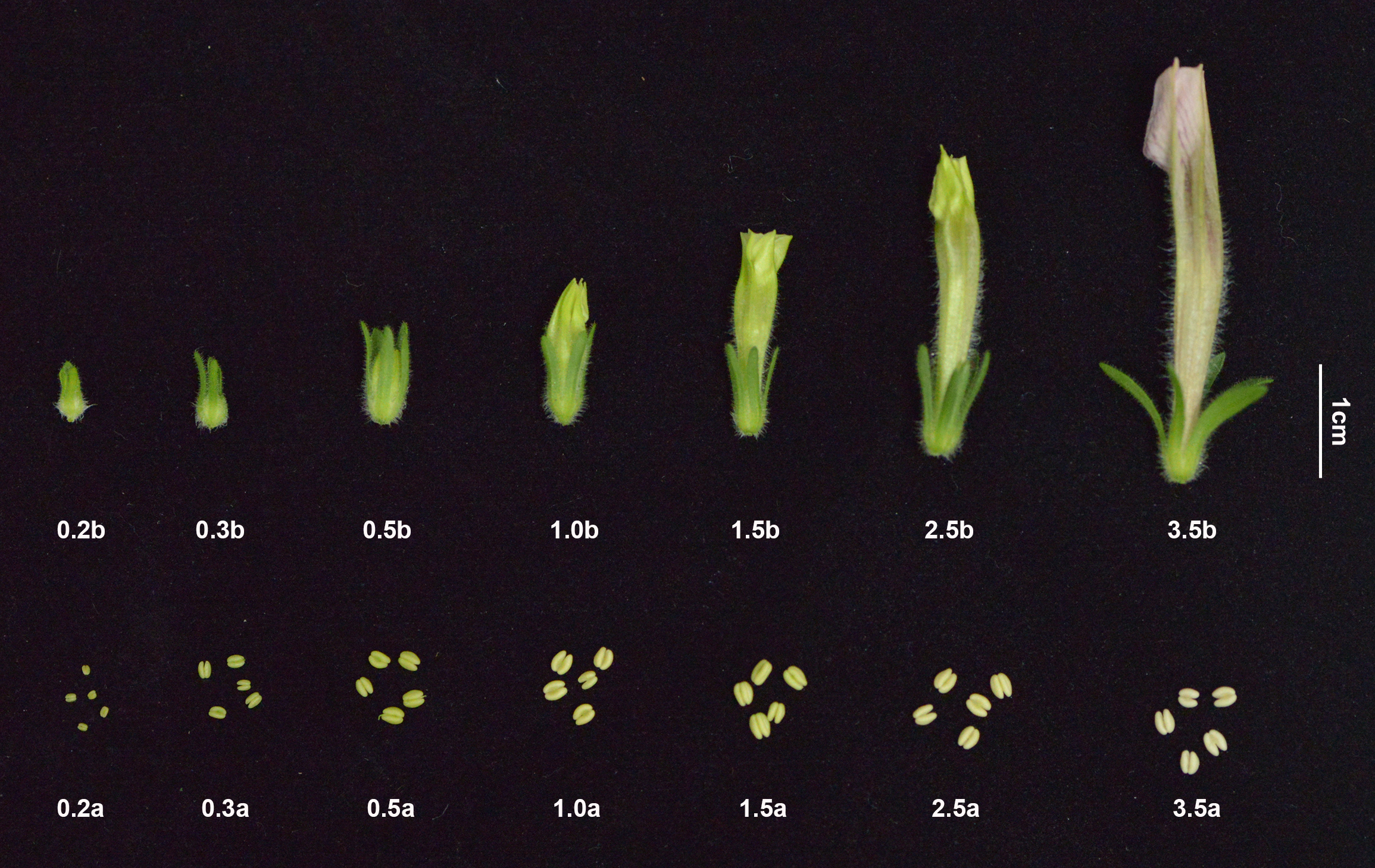


**Figure S1.** The seven developmental stages of flower buds (0.2b, 0.3b, 0.5b, 1.0b, 1.5b, 2.5b, and 3.5b) and anthers (0.2a, 0.3a, 0.5a, 1.0a, 1.5a, 2.5a, and 3.5a) based on flower bud length at 0.2, 0.3, 0.5, 1.0, 1.5, 2.5, and 3.5 cm (deviation range ±0.2 mm) in *Petunia hybrida* 'Fantasy Red'.
