# Supplementary material for: Genome-Wide Identification and Characterization of JAZ Protein Family in Two Petunia Progenitors
Source: Plants (Basel). 2019 Jul 3;8(7):203. doi: 10.3390/plants8070203 (PMC6681285; doi:10.3390/plants8070203)
Supplement: Supplementary file 1 [file plants-08-00203-s001.zip › Supplementary Materials-proofreading/Figure S2.docx]

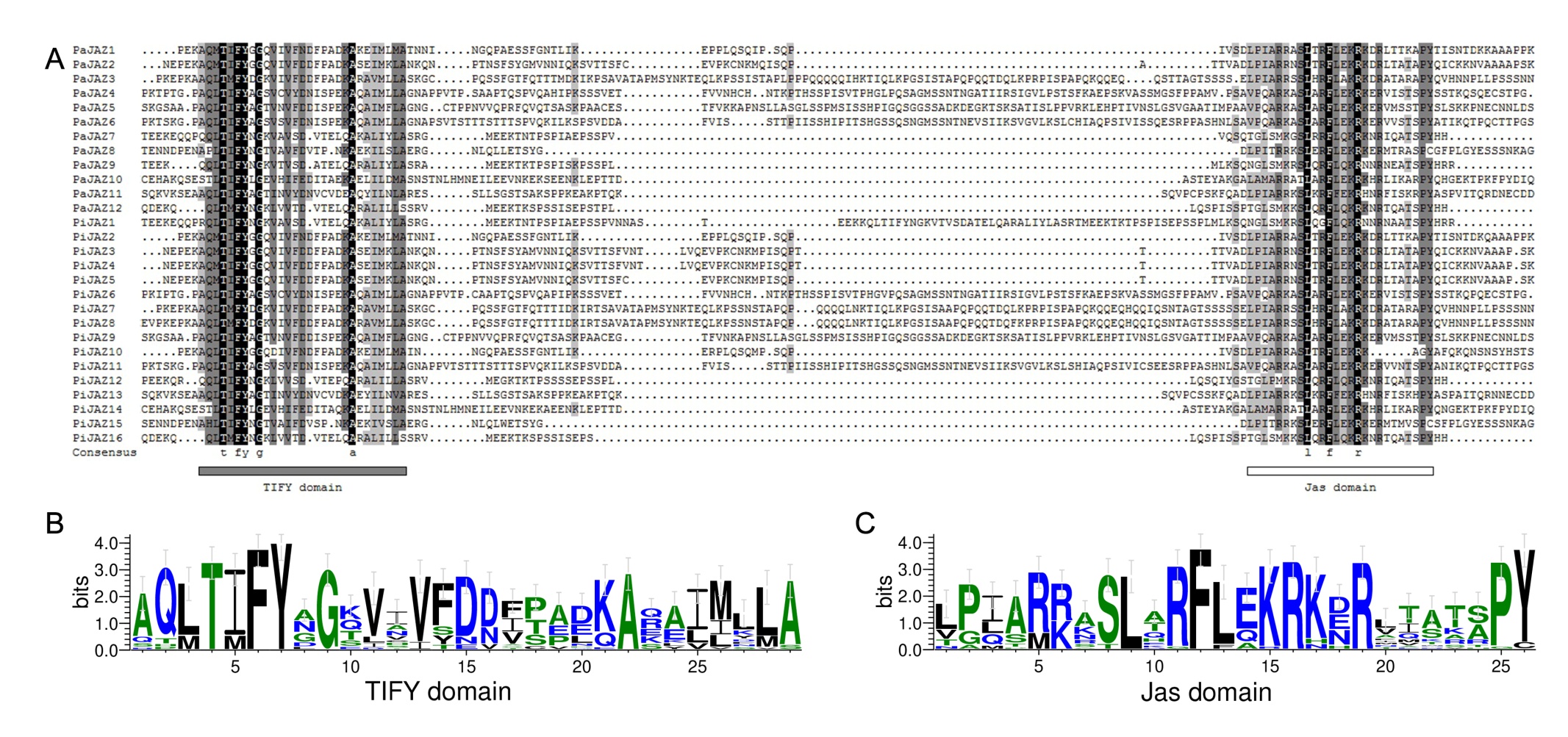
**Figure S2.** (**A**) The homologous sequence alignment of petunia JAZ family proteins. The sequence logos of the TIFY domain (**B**) and the Jas domain (**C**) were based on alignments of all petunia JAZ proteins, the bit score indicates the information content for each position in the sequence.
