# Supplementary material for: Genome-Wide Identification and Characterization of JAZ Protein Family in Two Petunia Progenitors
Source: Plants (Basel). 2019 Jul 3;8(7):203. doi: 10.3390/plants8070203 (PMC6681285; doi:10.3390/plants8070203)
Supplement: Supplementary file 1 [file plants-08-00203-s001.zip › Supplementary Materials-proofreading/Figure S3.docx]

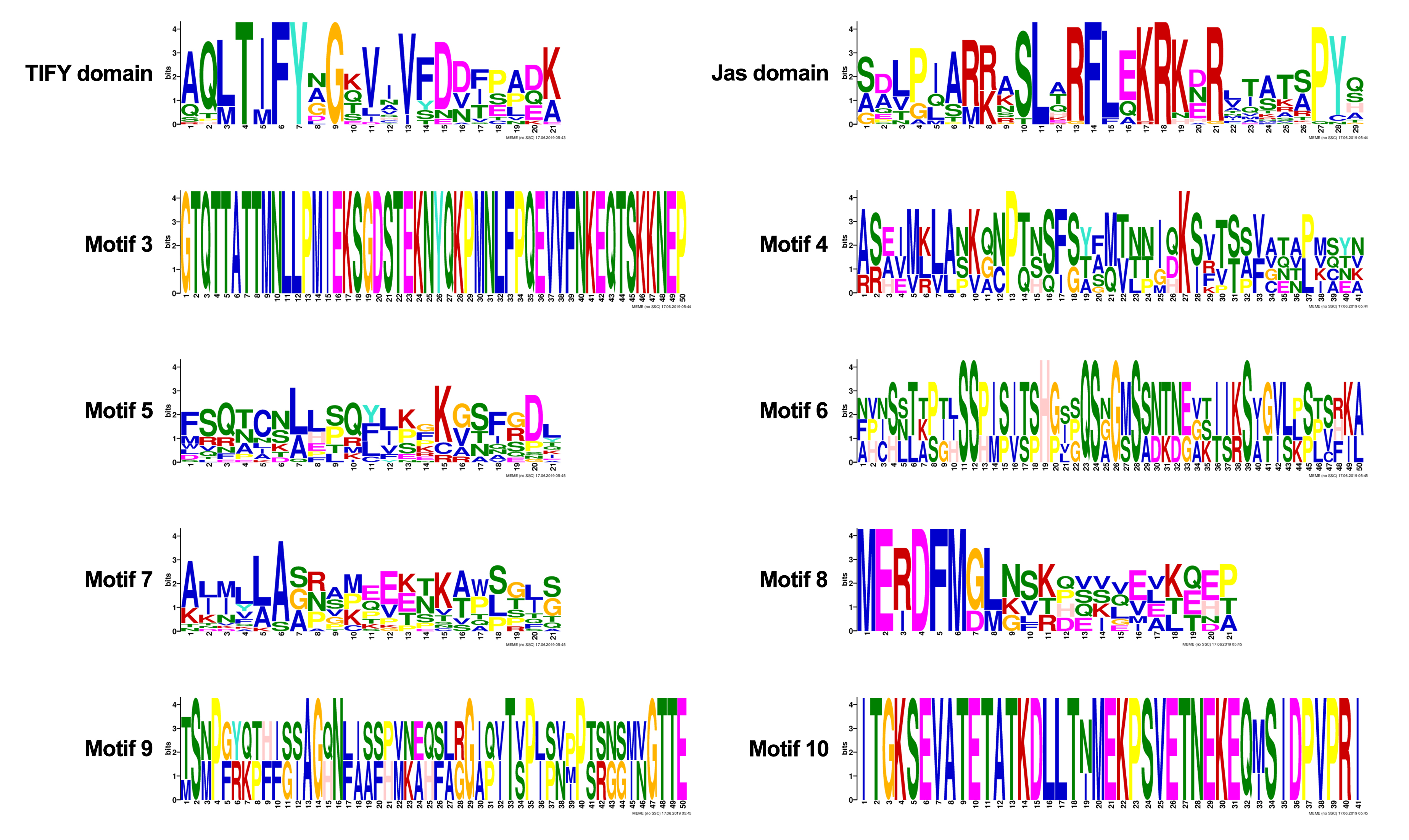


**Figure S3.** The sequence logos of the 10 motifs generated by MEME software. The bit score indicates the information content for each position in the sequence.
