# Supplementary material for: Genome-Wide Identification and Characterization of JAZ Protein Family in Two Petunia Progenitors
Source: Plants (Basel). 2019 Jul 3;8(7):203. doi: 10.3390/plants8070203 (PMC6681285; doi:10.3390/plants8070203)
Supplement: Supplementary file 1 [file plants-08-00203-s001.zip › Supplementary Materials-proofreading/Figure S4.docx]

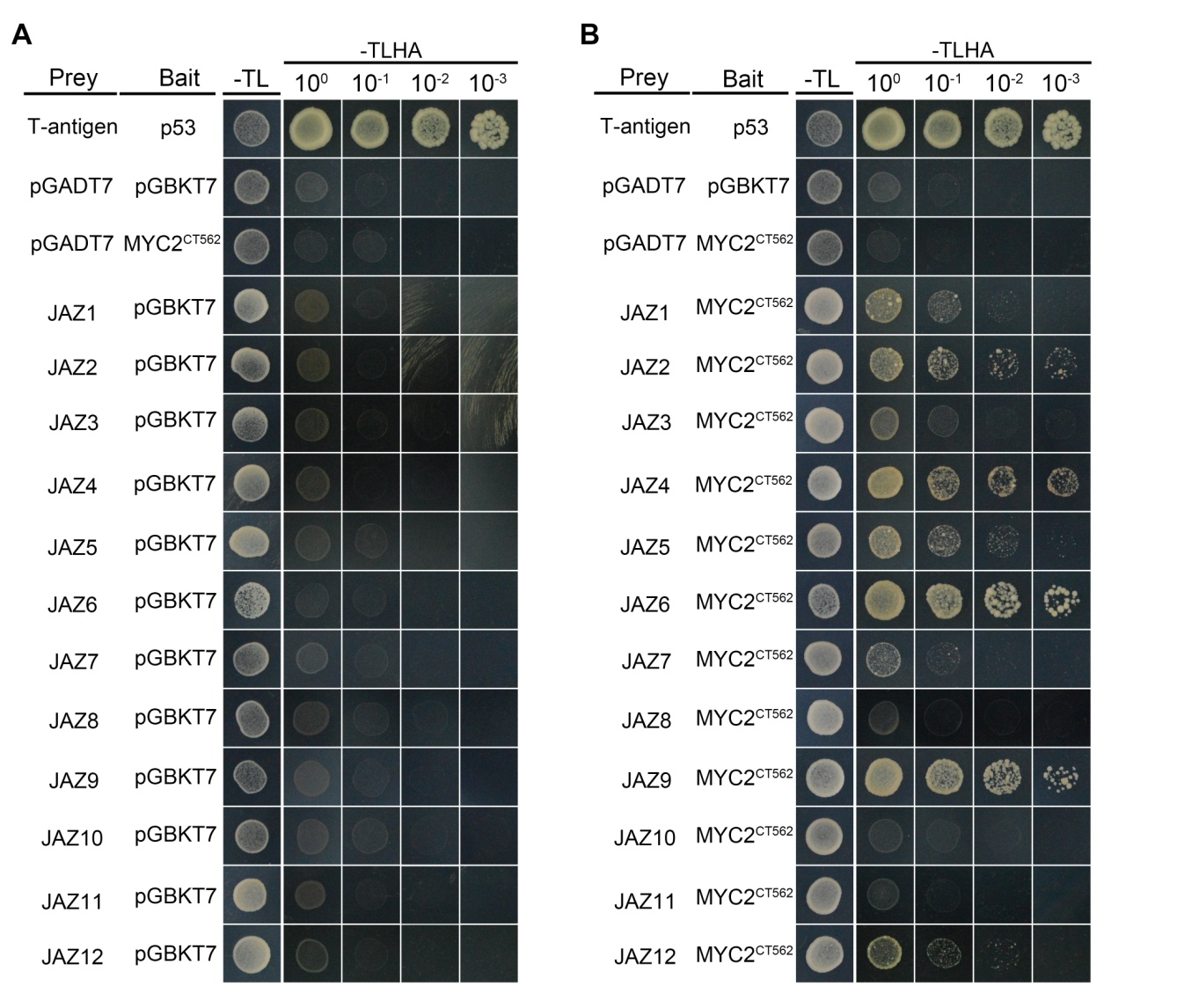


**Figure S4.** Y2H assays to test the interaction between PaJAZ proteins and transcription factor MYC2. Yeast strains AH109 carrying a combination of empty pGBKT7 (bait) and various pGADT7-PaJAZ constructs (prey) with a set of 10x dilutions were plated on solid synthetic dropout medium lacking Trp, Leu, His, and Ade (SD/-TLHA) as controls (**A**), and yeast strains carrying a combination of recombinant pGBKT7-MYC2^CT562^ (bait) and various pGADT7-PaJAZ constructs (prey) with a set of 10x dilutions were plated on SD/-TLHA medium to test protein interactions (**B**).
