# Supplementary material for: Genome-Wide Identification and Characterization of JAZ Protein Family in Two Petunia Progenitors
Source: Plants (Basel). 2019 Jul 3;8(7):203. doi: 10.3390/plants8070203 (PMC6681285; doi:10.3390/plants8070203)
Supplement: Supplementary file 1 [file plants-08-00203-s001.zip › Supplementary Materials-proofreading/Table S1.docx]

**Table S1.** The number and composition of *cis*-acting regulatory elements is in the promoter regions of *JAZ* genes in *P. axillaris*.

| **Gene name** | **element name** | | | | | | | | | |
| --- | --- | --- | --- | --- | --- | --- | --- | --- | --- | --- |
|  | CGTCA-motif ^1^ | TGACG-motif ^2^ | G-box ^3^ | WUN-motif ^4^ | ABRE ^5^ | ERE ^6^ | TCA-element ^7^ | LTR ^8^ | TC-rich repeats ^9^ | MBS ^10^ |
| *PaJAZ1* |  | 1 |  |  | 1 | 2 |  |  | 1 |  |
| *PaJAZ2* | 1 |  | 1 | 1 | 2 |  |  |  |  |  |
| *PaJAZ3* |  | 1 | 1 |  | 2 |  |  |  |  |  |
| *PaJAZ4* |  | 1 | 5 | 1 | 6 | 1 |  | 1 |  |  |
| *PaJAZ5* | 1 |  | 5 |  | 3 | 1 | 1 | 1 | 1 |  |
| *PaJAZ6* |  | 2 |  |  |  |  |  |  |  |  |
| *PaJAZ7* |  |  |  |  | 2 |  |  |  |  |  |
| *PaJAZ8* |  |  | 2 |  | 1 |  | 1 |  |  |  |
| *PaJAZ9* |  |  | 4 |  | 3 |  |  | 2 |  |  |
| *PaJAZ10* | 2 |  | 2 | 1 | 3 |  | 1 |  |  |  |
| *PaJAZ11* | 1 |  |  |  |  |  |  |  | 1 |  |
| *PaJAZ12* |  | 1 | 1 |  | 2 |  |  |  |  | 1 |

^1, 2^ cis-acting regulatory element involved in the MeJA-responsiveness; ^3^ MYC2 binding site; ^4^ wound-responsive element; ^5^ cis-acting element involved in the abscisic acid responsiveness; ^6^ ethylene responsive element; ^7^ cis-acting element involved in salicylic acid responsiveness; ^8^ cis-acting element involved in low-temperature responsiveness; ^9^ cis-acting element involved in defense and stress responsiveness; ^10^ MYB binding site involved in drought-inducibility.
