# Supplementary material for: Genome-Wide Identification and Characterization of JAZ Protein Family in Two Petunia Progenitors
Source: Plants (Basel). 2019 Jul 3;8(7):203. doi: 10.3390/plants8070203 (PMC6681285; doi:10.3390/plants8070203)
Supplement: Supplementary file 1 [file plants-08-00203-s001.zip › Supplementary Materials-proofreading/Table S2.docx]

**Table S2.** The primers used in this study.

| **Primer name** | **Forward sequence (5'→3')** | **Reverse sequence (5'→3')** | **Annotations** |
| --- | --- | --- | --- |
| qPaJAZ1 | GGGACACCCACTGCGACTAC | CCGCTGGCTGCCCATTA | qRT-PCR analysis |
| qPaJAZ2 | AGCCAGAAAAGGCACAAATGAC | TGGTGGGGTTTTGTTTGTTAGC |  |
| qPaJAZ3 | AAGAAGGACAGGAAAGGCGC | CTGTAGCGACTTCAGATTTGCC |  |
| qPaJAZ4 | CACAAGCACGCAAGGCATC | CAGGAGTGCTACATTCTTGGGAT |  |
| qPaJAZ5 | ATCGGTCAATCTGGTGGTAGTTCT | AATTCACAATCGTGGGATGCTC |  |
| qPaJAZ6 | TTCCAATCCTCCAACTCGCA | CAAAAACAGAAACTGAACCAGCATA |  |
| qPaJAZ7 | GGAATAACCAAAGCAATACCGAA | GCCTGAAGCTCTGTAACATCAGAA |  |
| qPaJAZ8 | TTCCCTCCATTGCCCCTTTA | CGAAAACAGCAACAGTCCCATT |  |
| qPaJAZ9 | CTTGTGCCTCCTTGTGTTTCCT | GCTGCTTCTCTTCTGTACTTGGGT |  |
| qPaJAZ10 | GGCTTCTAACTCCACCAATCTTC | GGACGCATCTGTTGTAGGCTCT |  |
| qPaJAZ11 | CCCAGCAAGTTTCAAGCAGAC | AGTAATCACAGGAGACGCATAAGG |  |
| qPaJAZ12 | CAATGGGAAACTTGTGGTTACTGA | AAAGGTGTTGATGGCTCTGAAAT |  |
| qPaActin | GTTGGACTCTGGTGATGGTGTG | CCGTTCAGCAGTGGTGGTG | Internal control |
| PaJAZ5-YFP | CGCGTCGACATGGAAAGAGACTTCATGGGG | TCCCCCGGGGGTCTCCTTTCCAACCGA | Subcellular localization |
| PaJAZ9-YFP | CGCGTCGACATGAGAAGGAACTGTAACTTGGAGC | TCCCCCGGGGCGACGATGATATGGAGAAGT |  |
| PaJAZ12-YFP | CGCGTCGACATGAGAAGGAACTGTAACTTGGAAC | TCCCCCGGGGTGATGATAAGGAGAAGTTGCT |  |
| PaJAZ1-AD | GACGTACCAGATTACGCTCATATGATGGCTTCATCAGAGATTGTGG | TCGATGCCCACCCGGGTTAAATTTGCTCAGTTTTTACTGGA | Construction of prey vectors |
| PaJAZ2-AD | gacgtaccagattacgctcatatgATGGGGTCATCGGAGATC | tcgatgcccacccgggCTAAAAGTATTGCTCAGTTTTCACT |  |
| PaJAZ3-AD | gacgtaccagattacgctcatatgATGTCTAATTTGCAAAATTCTTATG | tcgatgcccacccgggCTATAACTTGAAATTGAGATCAAGC |  |
| PaJAZ4-AD | gacgtaccagattacgctcatatgATGGAAAGAGATTTTATGGGTATG | tcgatgcccacccgggTCACGTCTCCTTGACCAAATT |  |
| PaJAZ5-AD | gacgtaccagattacgctcatatgATGGAAAGAGACTTCATGGGG | tcgatgcccacccgggCTAGGTCTCCTTTCCAACCGA |  |
| PaJAZ6-AD | GACGTACCAGATTACGCTCATATGATGGAGAGAGATTTCATGGGC | TCGATGCCCACCCGGGCTAACTGGTTGCAGGAAGAGC |  |
| PaJAZ7-AD | gacgtaccagattacgctcatatgATGACAAGAAACTGTAACTTGGAG | tcgatgcccacccgggCTAGTGATGATAAGGAGAAGTTGCT |  |

**Table S2.** *Cont.*

| **Primer name** | **Forward sequence (5'→3')** | **Reverse sequence (5'→3')** | **Annotations** |
| --- | --- | --- | --- |
| PaJAZ8-AD | gacgtaccagattacgctcatatgATGGCGAGATCAGCATTAGAACT | tcgatgcccacccgggCTAGTTCTTGTTTCCAGCTTTGTTG | Construction of prey vectors |
| PaJAZ9-AD | gacgtaccagattacgctcatatgATGAGAAGGAACTGTAACTTGGAGC | tcgatgcccacccgggCTAGCGACGATGATATGGAGAAGTT |  |
| PaJAZ10-AD | gacgtaccagattacgctcatatgATGGAATCAAGAATGGAGATAGAT | tcgatgcccacccgggTTAGCTTTCCCAATGGACG |  |
| PaJAZ11-AD | GACGTACCAGATTACGCTCATATGATGTATTGCAGTTCCAAACACG | TCGATGCCCACCCGGGTTAACTTCTTTTTGCCTTCAAACTA |  |
| PaJAZ12-AD | gacgtaccagattacgctcatatgATGAGAAGGAACTGTAACTTGGAAC | tcgatgcccacccgggTAGTGATGATAAGGAGAAGTTGCT |  |
| MYC2-BD | tcagaggaggacctgcatatgATGACAGACTATAGATTACCAACAATGA | cggccgctgcaggtcgacAGCAATTCTGGATGTCAATGCTA | Construction of bait vectors |
| MYC2^NT300^-BD | tcagaggaggacctgcatatgATGACAGACTATAGATTACCAACAATGA | cggccgctgcaggtcgacACTTCCTTGAACTGTATTTGAAGAATC |  |
| MYC2^CT350^-BD | tcagaggaggacctgcatatgTCAGTTCCTTCAATTAGTAATAGTACTA | cggccgctgcaggtcgacAGCAATTCTGGATGTCAATGCTA |  |
| MYC2^NT88^-BD | tcagaggaggacctgcatatgATGACAGACTATAGATTACCAACAATGA | cggccgctgcaggtcgacCCAGAATATAGCATACGTCCATGT |  |
| MYC2^CT562^-BD | tcagaggaggacctgcatatgCAATCGTCTGAAGCTGATTTCTC | cggccgctgcaggtcgacAGCAATTCTGGATGTCAATGCTA |  |

Note: letters underlined were the adapter sequences.
